# Effects of Hyperbaric Oxygen on Inflammatory Response to Wound and Trauma: Possible Mechanism of Action

**DOI:** 10.1100/tsw.2006.78

**Published:** 2006-04-03

**Authors:** Noori S. Al-Waili, Glenn J. Butler

**Affiliations:** Life Support Technologies, Inc., Chronic Wound Treatment and Hyperbaric Medicine Center, The Mount Vernon Hospital, Mount Vernon, NY 10550, USA

**Keywords:** hyperbaric, oxygen, nitric oxide, prostaglandin, cytokines, inflammation, wound

## Abstract

There is growing interest in expanding the clinical applications for HBO_2_ (hyperbaric oxygen therapy) into new medical and surgical fields. The pathophysiology of response towards wounds, infection, trauma, or surgery involves various chemical mediators that include cytokines, prostaglandins (PGs), and nitric oxide (NO). The beneficial role played by HBO_2_ in wound healing, carbon monoxide poisoning, decompression sickness, and other indications is well documented. However, the exact mechanism of action is still poorly understood. This review addresses the effects of HBO_2_ on PGs, NO, and cytokines involved in wound pathophysiology and inflammation in particular. The results of this review indicate that HBO_2_ has important effects on the biology of cytokines and other mediators of inflammation. HBO_2_ causes cytokine down-regulation and growth factor up-regulation. HBO_2_ transiently suppresses stimulus-induced proinflammatory cytokine production and affects the liberation of TNFα (tumor necrosis factor alpha) and endothelins. VEGF (vascular endothelial growth factor) levels are significantly increased with HBO_2_, whereas the value of PGE2 and COX-2 mRNA are markedly reduced. The effect of HBO_2_ on NO production is not well established and more studies are required. In conclusion, cytokines, PGs, and NO may play a major role in the mechanism of action of HBO2 and further research could pave the way for new clinical applications for HBO_2_ to be established. It could be proposed that chronic wounds persist due to an uncontrolled pathological inflammatory response in the wound bed and that HBO_2_ enhances wound healing by damping pathological inflammation (anti-inflammatory effects); this hypothetical proposal remains to be substantiated with experimental results.

## INTRODUCTION

Proper management of a chronic wound requires adequate oxygen delivery to the tissue, adequate protein, vitamins, minerals, nutritional factors, a moist environment, an appropriate inflammatory milieu, debridement, and correction of contributing medical diagnoses. In some patients, these conditions are achieved easily and the wound closes properly. However, in other patients, no healing can be obtained despite aggressive management of these conditions. Following injury, acute or chronic inflammation is evident as part of the body's defense against damaged cells and invading pathogens. The pathophysiology of responses following trauma involves various chemical mediators, which include cytokines, prostaglandins (PGs), and nitric oxide (NO) (Fig. 1).

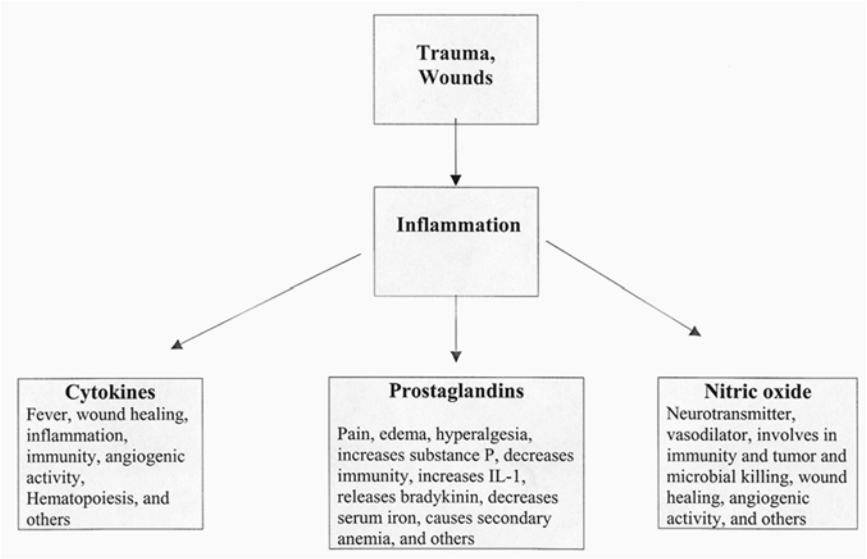

**FIGURE 1.** Mediators of biological response to trauma.

Current advances in adjunctive therapies such as $HBO_2$ (hyperbaric oxygen therapy), phototherapy, and alternative medicine, have enabled improved outcomes to be achieved. There is increasing interest in the beneficial role of $HBO_2$ in the field of wound healing[1,2,3,4]. $HBO_2$ means breathing pure (100%) oxygen under increased atmospheric pressure. $HBO_2$ induces high oxygen partial pressure in all tissues, reduces edema, causes activation of fibroblasts and macrophages, and stimulates angiogenesis and collage synthesis. $HBO_2$ chambers were originally developed at the turn of the 19th century to treat caisson workers and deep-sea divers who suffered from decompression sickness. The hyperbaric pressure can be delivered either with a monoplace chamber, which accommodates an individual patient usually in the supine position, or with a multiplace chamber, which will accommodate two or more patients. $HBO_2$ is safe and complications are uncommon.

Rarely, acute central nervous system toxicity including seizure may occur, usually in patients with predisposing conditions such as fever, head injury, or diabetes. At the treatment pressure commonly used for elective wound care (2.4 ATA), the incidence of oxygen seizure is 1.3/10,000 treatments for all patients. Pulmonary toxicity with pulmonary fibrosis and shortness of breath, which results from chronic exposure, are rarely seen during the maximum 20–30 treatment programs used for problem wounds. In the event that a patient develops symptoms, they are reversed by stopping treatment for a few days.

$HBO_2$ has shown promise in the management of chronic wounds. $HBO_2$ is thought to improve many aspects of poor healing by supplying high levels of oxygen at increased atmospheric pressure. It has been suggested that increasing the availability of oxygen does not necessarily stimulate the healing process, but that perhaps the pressure at which the oxygen is delivered is the responsible stimulus. The exact mechanism that explains the beneficial effect of $HBO_2$ on wound healing is not well understood. However, such an effect could be ascribed to the effects of $HBO_2$ on the inflammatory and immunological mediators. This article reviews scientific works describing the influence of HBO2 exposure on these mediators.

## CYTOKINES

Trauma and wounds induce an inflammatory response characterized by cytokine release and neutrophil activation and microvascular adherence[5,6]. Cytokines are polypeptides or glycoproteins, produced by macrophages and T-cells, that mediate and regulate immunity, inflammation, and hematopoiesis (Table 1). They act by binding to specific membrane receptors, which then signal the cell via secondary messengers to alter its biological activity. The receptors and their corresponding cytokines have been divided into several families based on their structure and activities. These include the hematopoietin, IFN (interferon), TNF (tumor necrosis factor), and chemokine family receptors. Responses to cytokines include increasing or decreasing expression of membrane proteins (including cytokine receptors), proliferation, and secretion of effecter molecules. Cytokines act over short distances and short time spans, and at very low concentration.

Cytokine is a general name; other names include lymphokine (cytokines made by lymphocytes), monokine (cytokines made by monocytes), chemokine (cytokines with chemotactic activities), and interleukin (IL) (cytokines made by one leukocyte and acting on other leukocytes). The largest group of cytokines that stimulate immune cell proliferation and differentiation includes IL-1, IL-2, IL-3, IL-4, IL-5, IL-6, IL-7, IFNγ, and granulocyte monocyte colony-stimulating factor.

IFN inhibits virus replication in infected cells and also stimulates antigen-presenting cells. Chemokines attract leukocytes to infection sites. Some cytokines, such as IL-10 and IL-12, are predominantly inhibitory and act on inflammatory cytokine production by macrophages. TNFα and IL-6 are proinflammatory cytokines. They enhance antimicrobial function and help tissue repair.

### Wound Healing and Cytokines

Wound healing, whether initiated by trauma, microbes, or foreign objects, shows an overlapping pattern of processes that include coagulation, inflammation, epithelialization, formation of granulation tissue, and matrix and tissue remodeling. These events are the main stages of wound repair: inflammatory, proliferative, and remodeling (Fig. 2).

Neutrophils release VEGF (vascular endothelial growth factor) and IL-8, which activate endothelial cells and induce angiogenesis by a paracrine feed-forward mechanism involving endothelial IL-8 up-regulation[7]. Neutrophils, however, are a rich source of proinflammatory cytokines, such as IL-8 and TNF[8]. IL-10 released from neutrophil fractions plays an active role in the development of post-traumatic immunosuppression[6] (Table 2).

The macrophages provide a continuing source of growth factors necessary to stimulate fibroplasia and angiogenesis, the fibroblasts produce the new extracellular matrix necessary to support cell in-growth, and blood vessels carry oxygen and nutrients necessary to maintain cellular metabolic process. Monocytes and macrophages express colony-stimulating factor 1, a cytokine necessary for the survival of monocytes and macrophages; TNFα, a potent inflammatory cytokine that causes fever, granulocytosis, and chemotaxis; and PDGF (platelet-derived growth factor), a potent chemoattractant and mitogen for fibroblasts. Other important cytokines expressed by monocytes and macrophages are TGFα (transforming growth factor), IL-1, TGFß, and insulin-like growth factor I[9]. Growth factors, especially PDGF and TGFß1 in concert with the extracellular-matrix molecules, presumably stimulate proliferation and migration of fibroblasts of the tissue around the wound, and express appropriate integrin receptors. Indeed, PDGF accelerates the healing of chronic pressure sores[10,11,12,13,14].

Many molecules have also been found to exhibit angiogenic activity, including VEGF, TGFß, angiogenin, angiotropin, and angiopoietin 1[15,16,17] (Table 1). Low oxygen tension and elevated lactic acid may also stimulate angiogenesis[18]. Activated epidermal cells of the wound secrete large quantities of VEGF[19]. FGF ( fibroblast growth factor) may initiate angiogenesis during the first 3 days of wound repair, whereas VEGF plays a role in angiogenesis during the formation of granulation tissue on days 4

through 7[20]. Proangiogenic cytokines include IL-1 and TNF, and antiangiogenic cytokines include IFN-γ and IL-12[21].

**TABLE 1**
**Cytokines and Their Functions on Wound Healing**

| Type of Cytokine | Function |
|---|---|
| Colony-stimulating factor 1 | Survival of monocytes and macrophages |
| Tumor necrosis factor α | Inflammatory cytokine<br>Activates macrophages<br>Stimulates angiogenesis<br>Stimulates synthesis of collagen and collagenase |
| Platelet-derived growth factor | Chemoattractant and mitogen for fibroblasts<br>Stimulates fibroblasts of the tissue around the wound<br>Accelerates the healing of chronic pressure sores<br>Stimulates remodeling and contraction<br>Chemotactic for most cells involved in wound healing (polymorphneuclocytes, fibroblasts, macrophages, smooth muscle cells)<br>Simulates angiogenesis<br>Activates wound healing cells |
| Transforming growth factor β1 | Stimulates fibroblasts of the tissue around the wound<br>Stimulates contraction<br>Attenuates ischemia-reperfusion injury<br>Inhibits apoptosis of myofibroblasts<br>Angiogenic activity |
| Transforming growth factor β2 | Stimulates fibroblast proliferation and the production of proteoglycans, collagen, and fibrin<br>Promotes accumulation of the extracellular matrix and fibrosis<br>Reduces scarring<br>Reverses the inhibition of wound healing by glucocorticoids<br>Induces contraction<br>Has angiogenic activity |
| Vascular endothelial growth factor | Angiogenic activity<br>Increases vasopermeability |
| Angiogenin, angiotropin, angiopoietin 1, thrombospondin | Angiogenic activity |
| Fibroblast growth factor | Stimulates wound contraction and epithelialization and production of collagen, fibronectin, and proteoglycans<br>Stimulates angiogenesis |
| IL-1 | Increases chemotaxis and collagen synthesis |
| IL-1, -2, -4, -5, and -6 | Stimulate macrophages and immune cells |
| IL-10 | Anti-inflammatory<br>Inhibits interferon and IL-2 secretion<br>Inhibits macrophage |
| IL-12 | Induces IL-2 production |
| Colony-stimulating factor | Stimulates hematopoiesis |
| Interferon γ | Stimulates macrophages and immune cells |
| Interferon α | Activates macrophages and regulates cytokines |
| Insulin-like growth factor I | Stimulates wound healing and fibroblast |
| Epidermal growth factor | The first cytokine described<br>Stimulates fibronectin synthesis, angiogenesis, fibroplasia, and collagenase activity<br>Stimulates keratinocyte migration and granulation tissue development. |

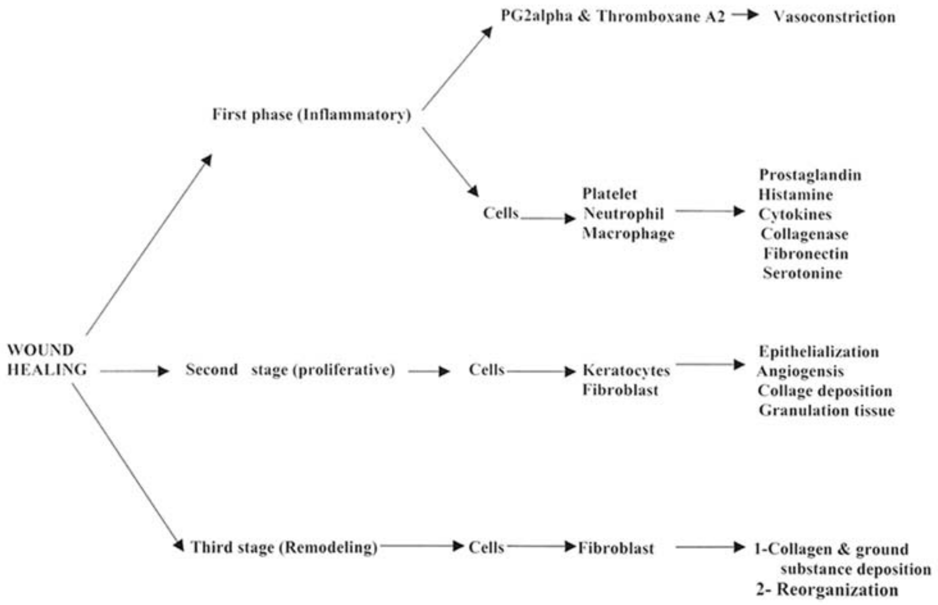

**FIGURE 2**. Stages of wound healing and cellular involvement.

**TABLE 2**
**Type of Cytokines Secreted by Various Cells Involved in Wound and Inflammation**

| Type of Cells | Cytokines Released |
|---|---|
| Macrophages | IL-1, -2, -6, -8, -12<br>TNF<br>FGF<br>EGF<br>TGFα and β<br>PDGF |
| Neutrophils | VEGF<br>IL-8<br>IL-1ra (inhibits IL-1)<br>TNF<br>IL-10 |
| Platelet | PDGF<br>TGFα and β<br>EGF |
| Mast cell | IL-1, -2, -6, -8<br>FGF |
| Endothelial cell | PDGF<br>TGF β<br>FGF |
| Lymphocyte cell | IFN α, β, γ<br>IL-1, -2, -6, -8, -10, -12<br>FGF<br>TGFα and β |
| Keratocyte cell | IL-1, -2, -6, -8<br>TGFα and β |
| Fibroblast | Keratocyte growth factor |

Wound contraction probably requires stimulation by TGFß1 or ß2, and PDGF; attachment of fibroblasts to the collagen matrix through integrin receptors; and cross-links between individual bundles of collagen[22,23,24]. Blockade of ß2-integrin signaling by the addition of antibodies against the CD11b to the cultures increased IL-10 production by macrophages from injured mice[25]. $HBO_2$ inhibits the function of human neutrophil ß2 integrins by a process linked to impaired synthesis of cGMP[26].

## Interaction Between PG, NO, and Cytokines in Wounds and Trauma

Various mediators such as PGs, NO, and cytokines are liberated following trauma and injury (Fig. 3). Macrophages isolated from injured mice produce higher levels of PGE2, TNFα, IL-6, and IL-10, and lower levels of IL-12 in response to lipopolysaccharide stimulation than do cells from sham-treated mice[25]. An early response to an acute inflammatory insult, such as in wound healing, is the conversion of arginine to the cytostatic molecule NO[27]. NO increases angiogenesis[28,29]. NO is implicated in angiogenesis induced by TGF and VEGF[30,31]. NO modulates chemoattractant cytokines including IL-8 and TGFß1[32,33]. NO stimulates the proliferation of endothelial cells, protects endothelial cells from apoptosis, and mediates VEGF production[34]. NO donors increase collagen formation in fibroblasts derived from both normal and wounded skin[35,36,37,38]. TNFα, IFNγ, and IL-1β enhance NO production by inducing the inflammation-associated biosynthetic enzyme[39]. IFNγ and IL-1β induce nitrite formation, and NO production and permeability; NO has been suggested to play a role in the induction of fibroblast apoptosis[40,41,42].

Exogenous PG of the E-series suppresses connective tissue proliferation[43]. COX-2 pathway plays a role in the regulation of the inflammatory phase of cutaneous wound repair[44,45]. Inhibition of this inflammatory pathway has also been suggested to reduce scar formation[44]. PGE2 augments collagen deposition and fibroblast proliferation in skin[46,47]. On other hand, PGE2 is known to reduce collagen deposition and fibroblast proliferation in the lung suggesting that PGE2 can have varied effects on cells depending on the type and origin of the cell[48,49,50]. NSAIDS, COX-1, and COX-2 inhibitors inhibit angiogenesis and delay wound and bone healing[51,52,53]. This might be due to inhibition of NO by these agents.

NO increases the activity of the COX-2 pathway in the setting of inflammatory cytokine stimulation. IL-1β induces PGE2 formation[42]. PGE2 and NO production are increased by TNFα, IL-1β, and IFNγ[54]. Both NO and PGE2 can have effects on fibroblasts in addition to modulating the contraction of collagen gels. PGE2 is a well-described inhibitor of fibroblast proliferation[55]. The production of PGE2 and NO induced by cytokines can affect fibroblast numbers as well as altering their contractile behavior.

## $HBO_2$ and Cytokines

$HBO_2$ affects the release of a number of cytokines and growth factors important to wound healing. Studies showed various effects of $HBO_2$ on cytokine production (Table 3). $HBO_2$ up-regulates FGF and collagen synthesis[56,57,58,59]. In patients with Crohn's disease, $HBO_2$ diminishes IL-1, IL-6, and TNFα levels[60]. $HBO_2$ effectively reduces heatstroke-induced plasma TNFα overproduction[61]. VEGF is up-regulated by hypoxia and hyperoxia of $HBO_2$[62]. The effects of TGFβ1 and PDGFβ are enhanced by $HBO_2$[63]. VEGF, TGFβ, and PDGFβ have biphasic release patterns; their release is stimulated by hypoxia and hyperoxia[64,65]. The activity of released VEGF is further enhanced during hyperoxia[65].

Human blood-derived monocyte-macrophages are stimulated before being transferred to an $HBO_2$ chamber where they were incubated in 97.9% $O_2$, 2.1% $CO_2$, at 2.4 ATA. It was found that a 90-min

$HBO_2$ exposure inhibits IL-1β by 23%, lipid A by 45%, phytohemagglutinin A by 68%, and TNFα by

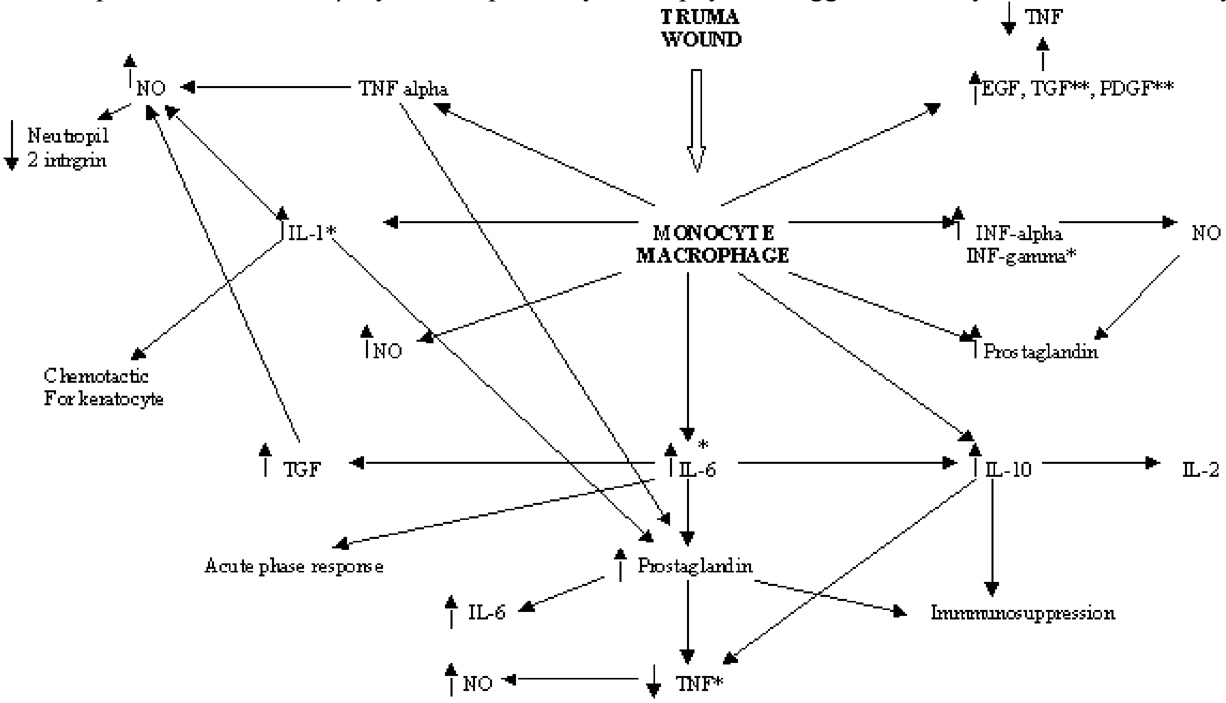

**FIGURE 3**. Interaction between cytokines, PGs, and NO. *Inhibited by $HBO_2$. **Elevated by $HBO_2$.

## TABLE 3
## Some Effects of $HBO_2$on Biological Mediators of Inflammation and Wound Healing

**Cytokines**

1. Enhance cytokine release
2. Decrease IFNγ release from stimulated lymphocytes
3. Increase endothelin 1, vasoconstriction
4. Decrease IL-1, IL-6, TNF
5. Increase VEGF level, angiogenesis
6. Decrease TNFα after ischemia reperfusion
7. Up-regulate FGF
8. Enhance release of TGFβ1 and PDGFβ

**PGs**

1. Decrease PGE2 in macrophages, bone, gingival, colitis, and kidney
2. Decrease COX-2 mRNA and protein production
3. Increase PGE2 in duodenal ulcer

**NO**

1. Increases NO production — increases angiogensis and inhibition of neutrophil 2–integrin function
2. Decreases NO production — causes vasoconstriction that could be prevented by ascorbic acid, superoxide dismutase, and prolonged $HBO_2$ exposure

27%. $HBO_2$ suppresses lipopolysaccharide-, lipid A–, and phytohaemagglutinin A–induced TNFα by 29, 31, and 62%, respectively. $HBO_2$ exposure transiently suppresses stimulus-induced proinflammatory cytokine production[66]. Van den Blink et al. found that $HBO_2$ enhances cytokine release of both unstimulated as well as lipopolysaccharide-challenged macrophages[67].

Another study examines the effects of hyperoxia, increased atmospheric pressure, and $HBO_2$ on cytokine synthesis in healthy volunteers who were exposed to 90 min of room air, 100% oxygen, 10.5% oxygen at 2 ATA, or 100% oxygen at 2 ATA $HBO_2$. Following the $HBO_2$ exposure, stimulated lymphocytes released 51% less IFNγ than cells obtained before the exposure. In addition, increased atmospheric pressure alone inhibited IFNγ secretion while room air and hyperoxia alone had no significant effect on IFNγ release[68]. $HBO_2$ in healthy volunteers can induce liberation of compounds such as TNFα and endothelins. It was suggested that liberation of endothelin 1 determines vasoconstriction occurring during $HBO_2$[69].

$HBO_2$ induces neovascularization in the partial-thickness skin graft while preserving regenerative capacity in the graft boundary and normal proliferative capacity of the epidermis[70]. Sheikh et al. reported that wound oxygen rises with $HBO_2$ from nearly 0 mmHg to as high as 600 mmHg[71]. The peak level occurs at the end of the 90-min treatment, and hyperoxia of lessening degree persists for approximately 1 h. The VEGF levels significantly increase with $HBO_2$ by approximately 40% 5 days following commencement of treatment. Increased VEGF production seems to explain in part the angiogenic action of $HBO_2$.

Treatment with $HBO_2$ ameliorates ischemia-reperfusion injury. Ischemia-reperfusion injury after transplantation leads to decreased bcl-2, an inhibitor of the apoptosis, and increased TNFα levels. TGFβ1, which is enhanced by $HBO_2$, ameliorates reperfusion injury by up-regulating bcl-2 and inhibiting TNFα and apoptosis of myofibroblasts[72]. $HBO_2$, at 2 ATA every other day, causes significant increases in bcl-2 and Mn-SOD immunoreactivity[73]. It was found that $HBO_2$ attenuates the increase in the TNFα and lung myeloperoxidase after intestinal ischemia reperfusion[74]. The number of neutrophils sequestered in the lung is reduced in $HBO_2$-treated rats compared to untreated rats. The results demonstrate that $HBO_2$ inhibits TNFα production during intestinal ischemia reperfusion.

Although $HBO_2$ has an immunosuppressive effect, it does not have any significant effect on phagocytotic activity[75,76,77]. However, a marked decrease in IL-1 and IL-2 production and a significant decrease in PGE2 production are observed[70,78]. IL-1 and IL-2 increase PG production. We have found that PGs are a potent immunosuppressive[79]. Therefore, the reduction of PG production may play an important role in the anti-inflammatory effect of $HBO_2$ and may ultimately result in enhanced local or general immune system. $HBO_2$ induces a significant increase in the spontaneous *ex vivo* secretion of TNFα by mononuclear cells from the rat blood, spleen, and lung[80]. Stimulation with lipopolysaccharides after exposure to $HBO_2$ induces a significant increase in TNFα secretion by lung and spleen macrophages compared with air controls.

Pretransplant tissue cultured in $HBO_2$ results in long-term allograft survival and the induction of systemic immune tolerance in a murine model[81]. It was shown that pretreatment of allogeneic stimulator cells with $HBO_2$ culture results in abrogation of cytotoxic T lymphocyte activity, proliferative responses, and IFNγ production[82]. In divers, the finding of a postdive increase in IL-6 suggests that an inflammatory response, probably created by a blood-gas interface, may be a factor in the development of DCI (decompression illness)[81]. $HBO_2$ decreased IL-6 and might explain, in part, its beneficial effects in divers. $HBO_2$ at 3 ATA increases the arterial oxygen tension over 2,000 mmHg, which should provide enough oxygen to tissues, even in the total absence of hemoglobin[80]. $HBO_2$ significantly attenuates decreases in arterial ketone body ratio after hemorrhage, with a significant reduction of mortality and cytokine induction.

## NITRIC OXIDE

NO plays important roles in diverse physiological and pathological processes, such as neurotransmission, vasodilatation, immunological response, wound repair, tumerogenesis, and inhibition of platelet aggregation[83,84]. NO has been proposed as a possible active agent for enhancing wound healing. In addition, NO increases cytosolic concentration of free calcium ions and it affects functions of various enzymes[84,85,86,87]. The activity of inducible NOS (nitric oxide synthase) is controlled at the level of gene transcription, whereas the activities of neuronal NOS and endothelial NOS are controlled by intracellular calcium/calmodulin, several different phosphorylation mechanisms, and by binding of the molecular chaperone heat shock protein 90. Low-molecular-weight thiols, albumin and hemoglobin, can carry NO in the blood stream[88,89,90].

### NO and Oxygen

Clinical experience with adjunctive $HBO_2$ therapy in the treatment of diabetic ulcers has shown that wound hyperoxia increases wound granulation, tissue formation, and accelerates wound contraction and closure. In addition to wound hyperoxia, increased wound NO production also appears to be important for successful diabetic wound healing. The impact of elevated $O_2$ tension on NO synthesis has not been clearly established by clinical studies. The possible elevation of NO concentration due to hyperoxia may contribute to the augmentation of angiogenesis and inhibition of neutrophil 2–integrin function that have been reported with $HBO_2$[91]. Endothelial cells, however, release superoxide anion, which is converted to $H_2O_2$ or reacts with NO to generate the strong oxidant, peroxynitrite. By reacting rapidly with NO, extracellular $O_2$ should decrease biologically available NO, which diffuses from the endothelium, erythrocytes, and vascular nerves to smooth muscle. Removal of $O_2$ from endothelial cells should prevent inactivation of NO by $O_2$ and increases its availability for vasodilation. Superoxide dismutase attenuates the degradation of NO.

Hyperoxic vasoconstriction is mediated by oxidative stress, which could be inhibited by vitamin C[92]. Elevated $O_2$ tensions above ambient will increase NO production by pulmonary endothelial cells and intact lungs[93,94,95]. In contrast, $O_2$ tensions above ~55 mmHg were reported to have little effect on NO production by cells obtained from the systemic circulation[96]. Furthermore, studies have demonstrated that hypoxic conditions diminish synthesis of NO in cells from both pulmonary and systemic circulations[93,94,97].

### Oxygen and Vasoconstriction

High arterial blood oxygen causes vasoconstriction in healthy humans[98,99,100]. Although hyperoxic vasoconstriction was first reported at least 90 years ago[98], the mechanism for this phenomenon in healthy humans is poorly understood. Several animal models of hyperoxic vasoconstriction suggest that $O_2$ tension may influence one or more of the endothelium-derived factors, such as NO, endothelin, and vasoactive PG[101,102,103]. It has been demonstrated that both hyperoxia and oxidative stress may stimulate increased production of the endothelium-derived vasoconstrictor endothelin[101,104].

### $HBO_2$ and NO

Previous studies have reported modulation of NOS activity by oxygen tension both *in vitro* and *in vivo* (Table 3). Pretreatment has beneficial hemodynamic effects in rats with endotoxin shock[94,105,106,107]. The beneficial effects of $HBO_2$ may be partially mediated by decreased NO production via reduced lipopolysaccharide-induced lung iNOS expression[108]. Another study used *in vivo* microdialysis to investigate the formation of oxygen-free radicals and NO in rat's brains under $HBO_2$ conditions. Male Sprague-Dawley rats exposed to 100% $O_2$ at a pressure of 3 ATA for 2 h, showed a six- and fourfold increase in nitrite/nitrate, in hippocampal and striatal dialysates, respectively. This increase was completely blocked by the NOS inhibitor L-nitroarginine methyl ester[109].

Another study demonstrates that exposures of rodents to oxygen at pressures of 2.0 and 2.8 ATA stimulates neuronal NOS and significantly increases steady-state NO concentration[110]. These studies demonstrate an increase in NO levels in response to $HBO_2$ treatment. One possible mechanism by which $HBO_2$ results in this increase is by increasing oxygen availability. The authors reported an increase in brain tissue $pO_2$ levels in response to $HBO_2$ treatment[111,112]. An increase in oxygen levels under $HBO_2$ conditions may, therefore, be a factor in increasing NO production under these conditions. Neuronal NOS activity contributes over 90% to total NO elevation due to hyperoxia. Cerebral cortex blood flow, measured by laser Doppler flow probe, is increased during hyperoxia and may be related to elevations of steady-state NO concentration[88]. It was found that relative lack of NO activity contributes to decreased cerebral blood flow under $HBO_2$, but, as exposure time is prolonged, NO production increases and augments regional cerebral blood flow[113].

It is well known that $HBO_2$ induces vasoconstriction in systemic organs, including the brain[114]. It was hypothesized that the cerebral blood flow is reduced by $HBO_2$ because of the inactivation of NO by superoxide anions[115]. Hyperbaric vasoconstriction was diminished after NO inhibition. Intravenous injection of superoxide dismutase increased the cerebral blood flow during air and $HBO_2$ exposure. These data suggest that inactivation of NO by superoxide anion is an effective mechanism of $HBO_2$ vasoconstriction[116]. The decreases in cerebral blood flow with $HBO_2$ are associated with a decrease in effective NO concentration and an increase in ROS(reactive oxygen species) production in the brain[116]. Studies on the central nervous system, however, have shown that an elevated partial pressure of $O_2$ increases concentration of NO by stimulating neuronal NOS activity[117]. Furthermore, prolonged $HBO_2$ exposure promotes NO production, which augments cerebral blood flow[88]. It has been shown that $HBO_2$ significantly inhibits the increase in plasma TNFα and NO induced by lipopolysaccharide treatment. $HBO_2$ corrects stress-impaired dermal wound healing and decreases iNOS expression associated with stress[118].

## PROSTAGLANDINS

PGs comprise a diverse family of lipid autocoids derived from cyclooxygenase-mediated metabolism of arachidonic acid, generating five primary bioactive prostanoids: PGE2, PGF2α, PGD2, PGI2, and thromboxane A2. The families of PGs and ILs are called eicosanoids. Cell membranes' phospholipids are converted to arachidonic acid by phospholipase A2. Lipoxgenase converts arachidonic acid to ILs; cyclooxygenase converts arachidonic acid to PGs; while epoxygenase converts arachidonic acid to DHETs(dihydroxyeicosatrienoic acid). PGs cause inflammatory pain, edema, and hyperalgesia; increase IL-6 production; increase NO production; cause release of tachykinine and increased substance P. PGs increased in the cells by direct interaction between NO and PGHs. They are generated in response to injury and inflammation and can sensitize or directly activate sensory endings of nociceptions.

It was found that the value of PGE2 in alveolar bone and gingiva is reduced markedly after $HBO_2$ exposure[119]. $HBO_2$ treatment causes a marked decrease in IL-1 production and a significant decrease in PGE2 production produced by splenic macrophages[78]. A marked inhibition of renal PGE2 excretion associated with antidiuresis effects during $HBO_2$ has been observed in a conscious dog[120].

The effect of $HBO_2$ on COX-2 expression after transient brain focal ischemia was evaluated. $HBO_2$ at 3 ATA for 1 h was administered at 6 h after reperfusion. The results showed that $HBO_2$ applied at 6 h after reperfusion significantly reduces infarct area as compared with a no-treatment group. $HBO_2$ decreases COX-2 mRNA and protein levels, which were up-regulated after ischemia reperfusion. Intervention with $HBO_2$ within 6 h reduces infarction[121].

The effect of a previous exposure to $HBO_2$ on the synthesis capacity of PGs and thromboxane was investigated in male rats' brains. Low-level hyperoxia significantly reduces the release of 6-keto-PGF1α and PGE2 in the striatum, without change in thromboxane B2[122]. Treatment by $HBO_2$ was accompanied by a significant decrease in colonic weight, PGE2 generation, myeloperoxidase, and NOS activities in experimental colitis[123]. PGE levels were measured in duodenal ulcer patients treated by H2-blockers of histamine receptors and $HBO_2$. Histadyl causes a marked decrease in gastric juice PGE concentrations in contrast to $HBO_2$ that raised PGE levels close to normal values[124].

## $HBO_2$ AND INFLAMMATION

The effect of $HBO_2$ on inflammation was studied experimentally in the treatment of experimental uveitis induced in rabbits[125]. The number of inflammatory cells and protein levels in the aqueous humor were reduced with use of $HBO_2$. Further, it was found that $HBO_2$ was comparable to corticosteroids in reducing inflammation. Animal studies of $HBO_2$ have shown that $HBO_2$ reduces infarct size and improving neurologic outcome, and inhibits inflammation and apoptosis after cerebral ischemia[126]. $HBO_2$ reduces ulcer depth and vascular thrombosis, and mortality in animals exposed to causative agent for esophageal injury[127]. $HBO_2$ ameliorates inflammatory changes and decreases dilatation of the intestine in animals subjected for muconeum peritonitis[128]. $HBO_2$ ameliorates the macroscopic damage and decreases plasma carbonyl content in trinitrobenzene sulfonic acid-induced chronic colitis in rats while it significantly reduces tissue myeloperoxidase activity in acute colitis[129]. $HBO_2$ markedly reduces the carrageenan-induced paw edema in rats by displaying anti-inflammatory activity[130]. In benign prostatic hyperplasia associated with inflammation, $HBO_2$ was found to be effective[131]. In severe acute pancreatitis, $HBO_2$ alleviates high spiking fever, improves white blood cell count and serum amylase levels, and reduces the abscess size[132]. Recently, we have found that $HBO_2$ helps to reduce edema and inflammation in compartment syndrome[133]. HBO2 had pulmonary protective effects during acute necrotizing pancreatitis[134]. These studies confirm preliminarily that HBO2 might possess anti-inflammatory properties.

## CONCLUSION

Studies on the effects of $HBO_2$ on inflammatory mediators may not only explain its mechanism of action, but also expand its clinical application. The main conclusion of this review is:

1. The angiogenic action of $HBO_2$ may be a result of increased VEGF and NO production.
2. The immunosuppressive effect of $HBO_2$ may be due to reduction of IL-1 production.
3. The anti-inflammatory effect of $HBO_2$ may be due to inhibition of IFNγ, PGs, TNFα, IL-1, and IL-6.
4. The beneficial effect on ischemia-reperfusion injury may be due to up-regulation of bcl-2 and TGF and reduction of neutrophil 2–integrin.
5. $HBO_2$ may stimulate local and general immunity because it decreases PGs, IL-1, and IL-10.
6. Vasoconstriction may be due to inhibition of NO and liberation of endothelin.
7 The anti-inflammatory effect of $HBO_2$ may stimulate the use of $HBO_2$ in acute or chronic inflammatory diseases in which large amounts of PGs are produced.
8. $HBO_2$ raises PGE levels close to normal values in patients with duodenal ulcer. This may encourage the use of $HBO_2$ in patients with duodenal or gastric ulcer.

Generally, the final conclusion regarding effects of $HBO_2$ on various mediators of inflammation is not fully documented and waits further clinical and laboratory investigation. Absolutely, conclusive results could pave the way for new applications of $HBO_2$.

## BIOSKETCH

**Noori S. Al-Waili**, **MD, DOG, PhD, CHT**, Director of Clinical Research, Life Support Technologies, Inc., Chronic Wound Treatment and Hyperbaric Medicine Center, The Mount Vernon Hospital, Mount Vernon, NY. Dr. Al-Waili is a physician and research scientist who has published more than 160 scientific papers in nephrology, urology, alternative medicine, hypertension, diabetes, malignancy, immunity, and biomedical sciences, including a wide range of new therapeutic measures. He has developed many theories and discoveries in modern and alternative medicine; published in esteemed international scientific journals; published several exciting papers in nephrology/urology including peritoneal macrophages transfusion, enuresis, renal colic, frequency of micturiton, and many papers on hyperbaric medicine and wound care; and more than 40 scientific clinical and laboratory publications on alternative medicine and honey. Presently, his main interest is focused on nephrology/urology, wound care, and hyperbaric medicine, as well as alternative medicine. He serves as an Editorial Board member of several medical journals, a member of evaluator panel, Current Drug, U.K., and a reviewer for many medical and biomedical journals.
